# Impact of Infection Control on Prevalence of Surgical Site Infections in a Large Tertiary Care Hospital in Haiphong City

**DOI:** 10.3390/antibiotics12010023

**Published:** 2022-12-23

**Authors:** Jérôme Ory, Quang Le Minh, Hung Phan Tien, Vinh Vu Hai, Elodie Careno, Tatiana Price, Alexandre Andrieux, Julien Crouzet, Catherine Dunyach-Rémy, Didier Laureillard, Jean-Philippe Lavigne, Albert Sotto

**Affiliations:** 1 Bacterial Virulence and Chronic Infections, INSERM U1047, University of Montpellier, 30029 Nîmes, France; 2 Department of Microbiology and Hospital Hygiene, CHU Nîmes, University of Montpellier, 30029 Nîmes, France; 3 Department of Hospital Management, Viet Tiep Hospital, Hai Phong 180000, Vietnam; 4 Department of Orthopedic Surgery, Viet Tiep Hospital, Hai Phong 180000, Vietnam; 5 Department of Infectious and Tropical Diseases, Viet Tiep Hospital, Hai Phong 180000, Vietnam; 6 Department of Infectious Diseases, CHU Nîmes, University of Montpellier, 30029 Nîmes, France; 7 Department of Infectious Diseases, CH Bagnols, 30200 Bagnols sur Cèze, France

**Keywords:** surgical site infection, Vietnam, antisepsis, infection control, healthcare-associated infection, point prevalence, antibiotic prophylaxis

## Abstract

Few point prevalence surveys (PPS) have been conducted in Vietnam on Surgical Site Infections (SSI) or antimicrobial use in surgery. The objective of this study was to evaluate the PPSs of SSI before and after implementation of antibiotic stewardship programs (ASP) and infection control (IC) in a Vietnamese tertiary care hospital. ASP and IC practices were implemented in operating rooms and the orthopedic department, including antibiotic training, skin preparation, hand hygiene, gloves and sterile instruments, and SSIs risk factors. A PPS of SSIs and antimicrobial use was performed in January 2016 according to methods from the Centers for Disease Control and Prevention, before ASP and IC, and in December 2019. Information recorded included surgical data, antibiotic prophylaxis, microorganisms, and SSI risk factors. Skin preparation compliance assessed preoperative washing and antisepsis. SSI prevalence was 7.8% in 2016 versus 5.4% in 2019 (*p* = 0.7). The use of prophylactic antibiotics decreased from 2016 to 2019. A third-generation cephalosporin was prescribed more than 48 h after surgery for most patients. Skin preparation compliance increased from 54.4% to 70.5% between assessments. The decreased SSI, although non-statistically significant, warrants continuing this program. Vietnamese hospitals must provide comprehensive IC education to healthcare workers to address the prevention of SSI and establish IC policies.

## 1. Introduction

Healthcare-associated infections (HAI) are the most frequently occurring medical adverse events in healthcare settings. Among 100 hospitalized patients, 7 in advanced countries and 10 in lower- and middle-income countries acquire an HAI [1]. Surgical site infections (SSI) are the most frequently reported types of HAIs [2]. Prevalence rates of SSIs range from 0.9% to 17.8% for all surgeries, although these are likely underestimates because many infections occur after patient discharge [1,3]. SSIs are associated with increased morbidity and mortality. Certain patients may also require reoperation, which is associated with prolonged hospitalization and rehabilitation. SSIs constitute a financial burden and negatively affect patient quality of life [4]. Finally, SSIs also contribute to antibiotic resistance through increasing exposure to broad-spectrum antibiotics, particularly for deep and prosthetic joint infections, which often require prolonged antibiotic treatment [5]. The severity of SSIs has led to an increased focus on SSI prevention [6,7], culminating with evidence-based recommendations to minimize the risk of preoperative, intraoperative, and postoperative infections by the World Health Organization (WHO) and the Centers for Disease Control and Prevention (CDC) [8]. One of the most important measures to prevent SSIs is the preoperative skin preparation during orthopedic surgery. The antiseptic and the techniques used to apply antiseptics may also influence the effectiveness in reducing SSI [5,9].

Prevalence surveillance is the reference standard for surveillance of HAIs, and many countries perform targeted HAI incidence surveillance, such as surveillance of SSIs [10,11]. In Southeast Asia, however, prevalence surveys of HAIs are not routinely performed [12]. Furthermore, few point prevalence surveys (PPS) have been conducted in Vietnam on either SSIs or on antimicrobial use. Indeed, the last PPS was conducted in 2008 in 36 Vietnamese hospitals [13]. Among 1230 surgeries, 223 patients had SSIs. In 2003, in Viet Tiep Hospital in Hai Phong (Vietnam), a PPS of SSI determined a prevalence around 15% (our unpublished data).

Optimizing the use of antibiotics is critical to effectively treat infections, protect patients from harms caused by unnecessary antibiotic use, and combat antibiotic resistance. The Ministry of Health in Vietnam developed a National Action Plan on antimicrobial resistance from 2013 to 2020. Antimicrobial stewardship programs (ASP) were listed as priorities in the plans in order to measure and optimize antimicrobial use and aim to slow the spread of resistant pathogens [3]. ASP can help clinicians improve clinical outcomes and minimize harm by improving antibiotic prescription [8].

Our study involved performing a PPS of SSI before and after implementation of ASP and infection control (IC) in a department of orthopedics in a large tertiary care hospital in Hai Phong city, Vietnam. The secondary outcomes were to describe the compliance with IC.

## 2. Results

### 2.1. Point Prevalence Survey (PPS)

A total of 88 patients were included (PPS1 = 51; PPS2 = 37). Their mean age was 44.2 years and 75% were male (Table 1). The prevalence of SSI was 7.8% (n = 4/51) and 5.4% (n = 2/37) for PPS1 and for PPS2, respectively (*p* = 0.70). Most patients were admitted for trauma-related injuries and required emergency surgery (Table 1). Significant differences were found between the periods for diabetes mellitus, NNIS score, and scheduled surgery. Prophylactic antibiotic use before surgery was 98% (n = 50/51) and 91.9% (n = 34/37) for PPS1 and for PPS2, respectively (Table 1). For antibiotic prophylaxis, a third-generation cephalosporin was given for longer than 48 h after surgery in the majority of cases in both periods (PPS1 = 51 (100%); PPS2 = 28 (75.7%)). The prophylactic antibiotic use (>48 h) reduced significantly between PPS1 and PPS2 (*p* < 0.05).

Of the SSI identified, the most frequently isolated microorganism was *Staphylococcus aureus* (n = 3/6). No other microorganism was identified. Bacteriology results were rarely available for SSI due to limited resources.

### 2.2. Risk Factors Associated with SSIs

None of the risk factors associated with SSI were significantly different between the groups with or without SSI, except general anesthesia, which was more common in SSI (9.8% vs. 50%, *p* = 0.024) (Table 2).

### 2.3. Implementation of IC and ASP

Thirty surgeons and residents were trained in IC practices and ASP in operating rooms and the department of orthopedics. In total, 19 and 17 surgeries were assessed, respectively, before and after implementation of prevention SSI protocols and training. These surgeries included the osteosynthesis material and removal of orthopedic implants for treatment of lower leg or scapula fractures. The antiseptic used was povidone-iodine. The first antiseptic application was performed for all surgeries. Overall compliance increased from 54.4% to 71% between the two assessments, particularly for preoperative washing, alcohol hand rubbing, rinsing, and drying (Table 3).

## 3. Discussion

The PPS is an important tool to prevent SSIs and to evaluate IC policies and educate physicians and nurses about this public issue [14]. In Viet Tiep hospital, the PPS of SSI patients hospitalized in the department of orthopedics found SSI rates of 7.8% and 5.4% between the two periods analyzed. In Southeast Asia, surveillance of HAIs is not a common practice. The lack of expertise and resources required for effective surveillance of HAIs limits the availability of data on the prevalence of SSIs. This study provides up-to-date information on the prevalence of these SSIs in Vietnam. The SSI rates of this study are higher than the rate in Asia, where the overall prevalence of SSI in clean and clean-contaminated surgeries was estimated at 4% (95% CI 4–5%) [4]. However, it is comparable to the rate in Vietnam in the last PPS from 2008 to 2010 (5.5%) [13]. The SSI rate was 15.8% for the open reduction of fracture and 0% for knee and hip prosthesis placement [13]. The patients presenting SSI in our study had all experienced emergency trauma. A high proportion of wounds with trauma operated in overcrowded surgical services were particularly dirty and contaminated, contributing to the high rate of SSI in this study (n = 72/88; 81.8%).

The SSI rate in orthopedics of 7.8% suggested inadequate IC practices, particularly in skin preparation before surgery. The skin antiseptic preparation was not systematically respected according to WHO and APSIC (Asia Pacific Society of Infection Control) recommendations [15]. Interestingly, the training and information posters improved the preoperative skin washing compliance from 21% to 70%, although no significant difference in the prevalence of SSI was noted. This improvement particularly affected the preoperative washing, alcohol hand rubbing, and rinsing and drying. Preoperative washing eliminates transient microbiota and some resident microbiota [5]. To improve training, an e-learning training program in Vietnamese has recently been created based on the skin preparation of the operation, surgical washing, and dressing repair [5]. This format allows caregivers to self-train. A new IC compliance evaluation will be necessary to estimate the impact of this training. To decrease microbial contamination in operating rooms, recommendations were implemented for surgeons and nurses. For example, the use of fans was prohibited during the surgical procedure. Posters prompted personnel to close the door. To limit the traffic in this room and to avoid opening during the surgery, a perioperative checklist was implemented (Supplementary Materials Figure S3) and a table was placed in front of the operating room door. There is a strong correlation between staff behavior (i.e., staff movements) and the number of door openings and levels of CFU/m3 in operating rooms [16,17]. A surveillance of environmental air and surveillance of surfaces in operating rooms could confirm the efficiency of these recommendations. The implementation of infection control decreased the prevalence of SSI, although non-statistically significantly. It should also be taken into account that the proportion of patients with risk factors (NNIS score, open wound, scheduled surgery, and diabetes mellitus) was significantly higher in the second period. In several studies, after the implementation of IC, a significant decrease in the prevalence of SSI has been observed, such as in Africa, Europe and the Unites States [18,19,20].

Another objective was to evaluate the impact of antibiotic stewardship in the department of orthopedics. In Vietnam, physicians have easy access to a wide variety of antimicrobials without restrictions. A third-generation cephalosporin was prescribed more than 48 h later in the majority of cases (60.2% (n = 53/88)) for the two periods. Preoperative antibiotic therapy was frequently administered to patients scheduled for clean elective surgery until the day of discharge (n = 84/88; 95.5%). Protocols of surgical antibiotic prophylaxis were developed between the two periods in terms of choice of drugs and duration. Asian guidelines suggest the use of narrow spectrum antibiotics within 1 h before incision, such as cefazolin for most surgical procedures, as surgical antimicrobial prophylaxis [15]. Postoperative antimicrobial therapy, defined as administration of antimicrobials staring more than 48 h after surgery (Prophylactic antibiotic (>48 h)), was frequently administered n = 79/88; 89.8%). The ECDC recommendations, based on a systematic review, are to not continue antibiotic prophylaxis after the end of surgery [21]. The postoperative antimicrobial therapy decreased significantly after the ASP (*p* < 0.05). However, the ASP is difficult to implement globally in emerging economies. Cost and human resources are the main impediments to implementing a successful ASP [22].

There are limitations of this study that should be taken into account. First, the study was conducted in only one orthopedic ward in Vietnam, and multicenter studies or national surveys are needed to quantify and monitor SSIs and antimicrobial use on a broader scale. Second, a greater species diversity should be found in these wounds. However, the identification of microorganisms in SSI in orthopedics is limited by the available technology and economic resources. Third, there was no patient follow-up, meaning that post-discharge SSIs could not be detected. The prolonged use of antibiotics in the postoperative period may decrease the incidence of, and the ability to detect, SSI. Fourth, the number of surgeries assessed was fairly low; however, different surgeons and different types of surgery were observed. Ongoing assessment should be implemented to improve surgical skin preparation practices.

## 4. Materials and Methods

### 4.1. Setting

This prospective non-interventional study was conducted from January 2016 to December 2019 at Viet Tiep Hospital in Hai Phong (Vietnam) and approved by the local Institutional Review Board (IRB number: 1085/QĐ-BVVT). Patient consent for inclusion was waived by the ethics body. This hospital is an acute care hospital located in the urban center of the city of Haiphong (population 2 million), third city in Vietnam. The Viet Tiep hospital is a public teaching hospital with 1140 beds, affiliated with Hai Phong University of medicine and pharmacy. It has a surgical ward covering the surgical specialties: digestive, neurosurgery, orthopedic, thoracic surgery, and urology. The orthopedic ward has 88 beds.

### 4.2. Point Prevalence Survey and Antimicrobial Use

Two PPSs of SSIs and of antimicrobial use were performed in the department of orthopedics in January 2016 (PPS1) and December 2019 (PPS2). The surveillance criteria were established by the CDC (CDC, 2012). All patients hospitalized in the recruiting departments for at least 48 h were evaluated and included. Information collected included patient demographics (sex, age, body mass index (BMI)), comorbidities, date of admission, presence of trauma, date of surgery, surgical diagnosis, and procedure. The National Nosocomial Infections Surveillance (NNIS [23]) risk score was calculated using American Society of Anesthesiologists (ASA) class (one point each for ASA score of 3, 4 or 5), operative time (operative time > 75th percentile of average of the surgery), and wound classification (contaminated, dirty or infected) [24]. SSI diagnosis was discussed between the committee composed of two infectious diseases specialists, one orthopedic surgeon, and one infection control practitioner, until reaching consensus. After bacterial isolation, the identification of microorganisms was performed by VITEK^®^-2 system (Biomérieux, Marcy l’Etoile, France). The nature and duration of antibiotic prophylaxis and curative antibiotic therapy were collected.

### 4.3. Implementation of Infection Control

IC practices in operating rooms and in the orthopedic department were assessed in surgeons and nurses from January 2016 to December 2019. In April 2018, hand sanitizer containing dethyl alcohol (Aniosgel 85NPC^®^, Lille-Hellemmes, France) was provided in wall-mounted dispensers in both locations. Hand sanitizer was available to healthcare workers, patients, and visitors. Once a year, healthcare workers were trained using posters, and an educational brochure explaining the importance of hand hygiene and when and how to clean hands was distributed on the wards. In April 2019, hand hygiene training was performed using ultraviolet cabinets for fluorescent-alcohol-based hand rubs. Surgical hand preparation, either by scrubbing with antimicrobial soap and water or alcohol-based hand rubs before donning sterile gloves, was improved by training using posters and an educational brochure.

In accordance with guidelines for the prevention of SSI (World Health Organization, 2019), the general surgery preparation procedure and the skin preparation procedure were implemented in operating rooms and the department of orthopedics. Posters in Vietnamese were hung in each room with the recommendations before the surgery, such as the preoperative shower. The skin preparation was performed in the operatory room using a sterile set for skin preparation containing the antiseptic, soap, sterile water, and three cups (Supplementary Materials Figure S1). The training for skin preparation consisted of recapping the different steps in orthopedic surgery (scrubbing and antisepsis) according to WHO recommendations [5].

Before and after implementation of SSI prevention protocols and training, the skin preparation for orthopedic surgery, whatever the orthopedic surgery (e.g., orthopedic implant, osteosynthesis), was assessed by the author (JO) on essential steps of skin preparation (World Health Organization, 2019). The preoperative washing was evaluated, comprising the depilation and preoperative shower. The four essential steps of skin preparation were also evaluated: rinsing, drying, and two antiseptic applications. Skin preparation compliance was determined (Supplementary Materials Figure S2). The behavior in the operatory room was noted by the author (JO) (door opening, window opening, traffic, and smoking).

### 4.4. Antibiotic Stewardship (ASP)

Once a year, training in antibiotic therapy and antibiotic prophylaxis for surgeons was provided by an infectious disease specialist on the orthopedic ward, in accordance with CDC guidelines [8]. The risk factors of SSIs were reminded (older age, obesity, smoking, diabetes mellitus, ischemia secondary to vascular disease or irradiation, and low serum albumin concentration). Protocols of surgical antibiotic prophylaxis were adapted in terms of drug choice and duration according to local microbial ecology. The local microbial ecology was determined by the microbiology lab, to identify the bacteria present in clinical sampling and their antibiotic susceptibility.

### 4.5. Statistical Analyses

Point estimates for rate of SSIs were computed. Fischer test exact (for categorical data) was performed to assess the relation between potential risk factors and outcome of interest. Results were expressed as a percentage or as mean ± standard deviation. The level of statistical significance was set at *p* < 0.05. Analyses were performed using GraphPad software version 9.4.1.

## 5. Conclusions

The decreased SSI, although non-statistically significant, warrants continuing IC practices and procedures in hospitals in lower- and middle-income countries. A local guideline for antimicrobial prophylaxis, empirical antimicrobial therapy, and directed use of antimicrobials is urgently needed. Future research in Vietnam should be conducted to assess the feasibility and potential impact of interventions to manage antimicrobial use, and to examine the financial burden of antimicrobial use and SSIs on patients and the healthcare system.

## Figures and Tables

**Table 1:** Characteristics of study population using the Point Prevalence Survey (PPS) performed in 2016 (PPS1) and in 2019 (PPS2).

	Total	PPS1 (n = 51)	PPS2 (n = 37)	*p* Value PPS1 vs. PPS2
Number of patients (n, %)	88	51	37	
Sex male (n, %)	66 (75.0)	41 (80.4)	25 (67.6)	0.2149
Age (years) (median, min–max)		45, min: 14–max: 74	47, min: 17–max: 77	
BMI (kg/m^2^) (median, min–max)		20.9, min: 15.9–max: 28.4	21.6, min: 16.6–max: 27.3	
ASA score (mean ± SD)		1.73 ± 0.57	1.56 ± 0.76	0.29
Emergency surgery (n, %)	72 (81.8)	45 (88.2)	27 (73.0)	0.09
Diabetes mellitus (n, %)	8 (9.1)	1 (2.0)	7 (18.9)	<0.0001
NNIS score (mean ± SD)		1.15 ± 0.63	0.64 ± 0.6	0.0025
Surgical Site Infections (n, %)	6 (6.8)	4 (7.8)	2 (5.4)	0.70
Open wound (n, %)	72 (81.8)	25 (49.0)	37 (100)	<0.0001
Drain (n, %)	42 (47.7)	26 (51.0)	16 (43.2)	0.52
Scheduled surgery (n, %)	43 (48.9)	17 (33.3)	26 (70.3)	0.032
General anesthesia (n, %)	12 (13.6)	6 (11.8)	6 (16.2)	0.75
Antibiotic used before surgery (n, %)	84 (95.5)	50 (98.0)	34 (91.9)	0.30
Prophylactic antibiotics (n, %)	84 (95.5)	50 (98.0)	34 (91.9)	0.30
Prophylactic antibiotics (>48 h) (n, %)	79 (89.8)	51 (100)	28 (75.7)	0.0002
Cumulative antibiotics (n, %)	31 (35.2)	19 (37.3)	12 (32.4)	0.66

BMI, Body Mass Index; NNIS, National Nosocomial Infections Surveillance; ASA, American Society of Anesthesiology.

**Table 2:** Risk factors associated with surgical site infection (SSI) in orthopedic patients.

	No SSI (n = 82)	SSI (n = 6)	*p* Value
Emergency trauma (%)	66 (80.5)	6 (100)	0.391
Average NNIS Score	0.93	1	0.9893
Open wound (%)	59 (71.9)	3 (50)	0.355
Drain (%)	33 (40.2)	4 (66.7)	0.4192
General anesthesia (%)	8 (9.8)	3 (50)	0.024
Antibiotic used before surgery (%)	72 (87.8)	6 (100)	0.99
Prophylactic antibiotics (%)	74 (90.2)	6 (100)	0.99

NNIS, National Nosocomial Infections Surveillance.

**Table 3:** Compliance with the criteria assessed on the skin preparation during orthopedic surgery.

	Assessment 1 (n = 19)	Assessment 2 (n = 17)
Preoperative washing	21.1% (n = 4/19)	70.6% (n = 12/17)
Alcohol hand rubbing before putting on gloves	22.2% (n = 4/18)	50% (n = 8/16)
Debridement	42.1% (n = 8/19)	71.4% (n = 10/14)
Rinsing	42.1% (n = 8/19)	71.4% (n = 10/14)
Drying	38.9% (n = 7/18)	60% (n = 9/15)
First antiseptic application	100% (n = 18/18)	100% (n = 17/17)
Second antiseptic application	73.7% (n = 14/19)	70.6% (n = 12/17)
Compliance with the criteria assessed	54.4% (n = 80/147)	70.5% (n = 91/129)

## Data Availability

The datasets used and/or analyzed during the current study are available from the corresponding author on reasonable request.

## Supplementary Materials

The following supporting information can be downloaded at: https://www.mdpi.com/article/10.3390/antibiotics12010023/s1, Figure S1: A sterile set for skin preparation implemented in operatory room including, the antiseptic (yellow bottle), the soap (pink bottle), the sterile water (clear bottle), the gloves, and three cups for skin preparation. The sterile wipe is not present in the picture. Figure S2: Assessment of the preoperative skin preparation. Figure S3: A perioperative checklist assessed the preoperative shower, the medical and surgical treatment, and clinical data.
