# Supplementary material for: Impact of Infection Control on Prevalence of Surgical Site Infections in a Large Tertiary Care Hospital in Haiphong City
Source: Antibiotics (Basel). 2022 Dec 23;12(1):23. doi: 10.3390/antibiotics12010023 (PMC9854418; doi:10.3390/antibiotics12010023)
Supplement: Supplementary file 1 [file antibiotics-12-00023-s001.zip › antibiotics-2069226-supplementary.pdf]

Figure S1: A sterile set for skin preparation implemented in operatory room including, the antiseptic (yellow bottle), the soap (pink bottle), the sterile water (clear bottle), the gloves and three cups for skin preparation. The sterile wipe is not present in the picture.

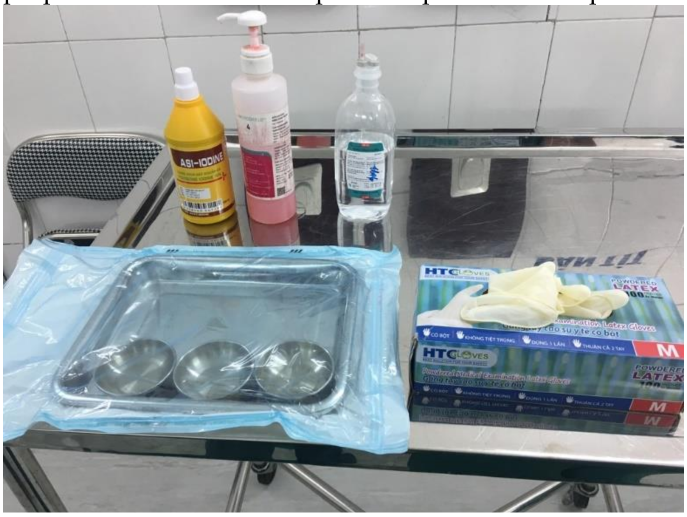

Figure S2: Assessment of the preoperative skin preparation
Record ID

Date……………………….. **Auditor………………………………..**

Surgical specialty:

- o Orthopedic
- o Digestive
- o Vascular
- o Urology
- o Ophthalmology
- o Oral Surgery
- o Plastic surgery
- o Neurosurgery

Number of surgical room:

Patient name:

Sex:

Age:

Kind of surgery:

- o Programmed
- o Programmed (< 24 h)
- o Emergency

Intervention

- o Hip prosthesis
- o Knee prosthesis
- o Osteosynthesis material
- o Other

Septic surgery

- o Yes No

Depilation//Preoperative washing

Has the patient undergone depilation?

- o Yes
- o No
- o Not necessary

Where was this depilation performed?

- o At home
- o In service
- o Operating room
- o Preparation room

How was it done?

- o Clipper
- o Chemical depilation
- o Shaver
- o Other:

Did the patient have a pre-operative shower?

o Yes No

When?

- o The day before
- o The morning

Before skin preparation

How was hand hygiene performed before wearing gloves?

- o Completed and compliant
- o Completed but not compliant
- o Not carried out

Debridement

How was debridement carried out on the operating site?

- o With non-sterile gloves
- o With sterile gloves

Which product was used?

- o PVPI scrub
- o Chlorhexidine scrub
- o Mild soap
- o Other

Was rinsing performed?

o Yes No

Was drying performed?

o Yes No

Were gloves changed after cleaning?

o Yes No

How was hand hygiene performed upon removing gloves?

- o Completed and compliant
- o Completed but not compliant
- o Not carried out

Antiseptis

Were sterile gloves used for the first antiseptic application of the operating?

o Yes No

Who performed the first antiseptic application?

o Surgeon

o Operating aid

o Instrumentalist/Nurse

o Circulating

Which product was used?

o Povidone-iodine (PVP-I)

o Alcoholic PVPI

o Alcoholic chlorhexidine

o Other

Was the drying time respected?

o <30 s

o >30 s

Was the wide operative field respected?

o Yes No

Who performed the second antiseptic application?

o Surgeon

o Operating aid

o Instrumentalist/Nurse

o Circulating

Was the second antiseptic application performed?

o Yes No

Were sterile gloves used for the second antiseptic application?

o Yes No

Which product was used?

Povidone iodine

Povidone iodine alcohol

Chlorhexidine alcohol

Other antiseptic

Was drying time respected?

<30 s

> 30 s

Did the antiseptic drips be noticed?

Yes No

Figure S3: A perioperative checklist assessed the preoperative shower, the medical and surgical treatment and clinical data

Figure S3a : Vietnamese version

Ngày:

Chuẩn bị mổ ngày:

- Giải thích bệnh nhân và gia đình
- Ký cam đoan mổ
- Hoàn thiện hồ sơ xét nghiêm

- Vệ sinh vùng mổ
- Thụt tháo phân nếu cần: - Có - Khong
- Ngủ sớm, nhịn ăn sáng ngay phẫu thuật
- Khám tiền mê trước phẫu thuật
- Đo Mạch, nhiệt độ, huyết áp sáng ngày phẫu thuật
- …….

Figure S3b: English version

Date:

Date of surgery :

- Explain to patient and family
- Sign a commitment to surgery
- Completing the examination file
- Cleaning the surgical area
- Enema if necessary: Yes No
- Sleep early, fast for breakfast right after surgery
- Pre-surgery pre-anesthesia examination
- Measure pulse, temperature, blood pressure on the morning of surgery
- ….
